# An *In Vitro* Study on Extracellular Vesicles From Adipose-Derived Mesenchymal Stem Cells in Protecting Stress Urinary Incontinence Through MicroRNA-93/F3 Axis

**DOI:** 10.3389/fendo.2021.693977

**Published:** 2021-08-16

**Authors:** Lu Wang, Yali Wang, Yuancui Xiang, Jinping Ma, Hui Zhang, Jingfang Dai, Yanan Hou, Yupei Yang, Jingru Ma, Hongjuan Li

**Affiliations:** Department of Obstetrics and Gynecology, Zhengzhou Central Hospital Affiliated to Zhengzhou University, Zhengzhou, China

**Keywords:** stress urinary incontinence, adipose-derived mesenchymal stem cells, extracellular vesicles, microRNA-93, coagulation factor III, fibroblasts, satellite cells

## Abstract

Since the potential roles of extracellular vesicles secreted by adipose-derived mesenchymal stem cells (ADSCs) are not well understood in collagen metabolism, the purpose of this research was to evaluate the effects of ADSCs-extracellular vesicles in stress urinary incontinence and the regulatory mechanism of delivered microRNA-93 (miR-93). ADSCs were isolated and cultured, and ADSCs-extracellular vesicles were extracted and identified. Stress urinary incontinence primary fibroblasts or satellite cells were treated with ADSCs-extracellular vesicles to detect the expression of Elastin, Collagen I, and Collagen III in fibroblasts and Pax7 and MyoD in satellite cells. After transfecting ADSCs with miR-93 mimics or inhibitors, extracellular vesicles were isolated and treated with stress urinary incontinence primary fibroblasts or satellite cells to observe cell function changes. The online prediction and luciferase activity assay confirmed the targeting relationship between miR-93 and coagulation factor III (F3). The rescue experiment verified the role of ADSCs-extracellular vesicles carrying miR-93 in stress urinary incontinence primary fibroblasts and satellite cells by targeting F3. ADSCs-extracellular vesicles treatment upregulated expression of Elastin, Collagen I, and Collagen III in stress urinary incontinence primary fibroblasts and expression of Pax7 and MyoD in stress urinary incontinence primary satellite cells. miR-93 expression was increased in stress urinary incontinence primary fibroblasts or satellite cells treated with ADSCs-extracellular vesicles. Extracellular vesicles secreted by ADSCs could deliver miR-93 to fibroblasts and then negatively regulate F3 expression; ADSCs-extracellular vesicles could reverse the effect of F3 on extracellular matrix remodeling in stress urinary incontinence fibroblasts. miR-93 expression was also increased in stress urinary incontinence primary satellite cells treated by ADSCs-extracellular vesicles. Extracellular vesicles secreted by ADSCs were delivered to satellite cells through miR-93, which directly targets F3 expression and upregulates Pax7 and MyoD expression in satellite cells. Our study indicates that miR-93 delivered by ADSCs-extracellular vesicles could regulate extracellular matrix remodeling of stress urinary incontinence fibroblasts and promote activation of stress urinary incontinence satellite cells through targeting F3.

## Introduction

Stress urinary incontinence (SUI), the most prevalent type of urinary incontinence, is defined as the involuntary leakage of urine on sneezing or coughing, or on effort or exertion (1). The most important factors of SUI in women are pregnancies, obesity, and menopause, while the factors that may trigger the onset of this disease in men are mainly related to surgical iatrogenic lesions (2). SUI not only profoundly damages people’s quality of life but also imposes a financial burden on the healthcare system (3). Pelvic floor muscle training is considered as the first-line treatment of SUI due to its absence of adverse effects (4), and continence support pessaries together with pharmacotherapy may also be prescribed to alleviate the symptoms of SUI (5). Nevertheless, because of poor long-term patient compliance, the therapeutic efficacy of these strategies is restricted (6).

Adipose-derived mesenchymal stem cells (ADSCs) have been widely studied due to their advantages of huge storage, safe and security, high speed of proliferation, easy to isolate, and low immunogenicity (7). In recent years, many pre-clinical and clinical studies have been published on stem cells for SUI treatment. For instance, Zou et al. have found that the tissue-engineered sling with bone marrow-derived mesenchymal stem cells (BMSCs) have presented good effects for SUI treatment in a rat model (8). In addition, Zhou et al. have also supported that ADSCs are able to reduce the abnormal voiding rate in a rat model of SUI (9). All these evidences indicate the regenerative potential of stem cells for SUI therapy. Extracellular vesicles (EVs), secreted from various cells, are small intraluminal vesicles delivering the intracellular contents, including noncoding RNAs, microRNAs (miRs), messenger RNA (mRNA), and cellular proteins (10). miRs act as negative modulators of gene expression *via* suppressing mRNA translation or triggering mRNA degradation (11). Evidence has shown that ADSCs-EVs can carry miR-93 (12), and role of miR-93 in the novel treatments for SUI has also been demonstrated (13). Notably, Jing et al. have reported that miR-93 modulates collagen loss through targeting stromelysin-1 in human nucleus pulposus cells (14). Therefore, it was speculated that miR-93 may affect collagen expression in SUI. Additionally, miRNAs are able to control gene expression posttranscriptionally *via* interacting with the specific target mRNAs 3’untranslated region (UTR) (15). An article has indicated that miR-93 directly targets coagulation factor III (F3) to participate in the pathogenesis of leiomyomas (16). Tissue factor (F3) is a cell surface receptor for the coagulation factor VII/VIIa, which is initially found as an initiator of the extrinsic coagulation pathway (17). However, the binding relationship between miR-93 and F3 in SUI needs further confirmation. In this study, we obtained high-purity EVs from rat ADSCs to figure out the *in vitro* therapeutic effects of ADSC-derived EVs in SUI with the involvement of miR-93 and F3.

## Materials and Methods

### Ethics Statement

This study was approved by the Animal Care and Use Committee of Zhengzhou Central Hospital Affiliated to Zhengzhou University. Postoperative animal care and therapeutic surgical interventions were conducted in accordance with the National Institutes of Health’s Laboratory Animal Care and Use Guidelines.

### Experimental Animals

Male Sprague-Dawley rats (8 weeks, 300–350 g) were available from SLAC Laboratory Animal Co., Ltd. (Shanghai, China). These rats were separately fed in the ventilated cages at the temperature of (24–26)°C and constant humidity, with a 12-h day/night cycle.

### Isolation and Identification of ADSCs

The rats were intraperitoneally injected with 2% pentobarbital sodium for anesthesia, and then euthanized and soaked in 75% alcohol for 10 min. The subcutaneous adipose tissues in bilateral groin were harvested from rats, rinsed with phosphate-buffered saline (PBS), and detached with 0.2% collagenase I (Sigma-Aldrich, St. Louis, MO, USA) for 1 h at 37°C thermostatic waterbath. Next, the detached tissues were rinsed with Dulbecco’s modified Eagle’s medium (DMEM, Sigma-Aldrich, St Louis, MO, USA) supplemented with 15% fetal bovine serum (FBS, Gibco BRL, Maryland, USA), followed by centrifugation at 1,200 g for 10 min to discard the mature adipocytes. Subsequently, the pellet was resuspended in DMEM containing 15% FBS, 100 U/ml penicillin and 100 μg/ml streptomycin, and then cultured at 37°C under the conditions of 5% CO_2_. ADSCs were detached several times with 0.02% ethylenediaminetetraacetic acid (EDTA)/0.25% trypsin (Sigma-Aldrich) for 5 min. When reaching 80–90% confluence, ADSCs were detached with 0.25% trypsin for passage.

Identification of ADSCs by flow cytometry: the ADSCs at passage 3 were detached with 0.25% trypsin and divided into 10 EP tubes to prepare 100 μl single cell suspension with a cell density of 1 × 10^6^ cells/ml. The first five tubes were set as negative controls (NCs), and the same amount of the corresponding isotype control antibody was added. The remaining five tubes were supplemented with 1 μl phycoerythrin (PE)-conjugated CD31, CD34, CD29, CD90, and CD105 (BD Bioscience, San Jose, CA, USA) and incubated for 15–20 min devoid of light. Next, the cell suspension was suspended with 400 μl PBS and detected on a flow cytometer (caliber, BD, USA) (18).

ADSC osteogenic and adipogenic differentiation potential: ADSCs at passage 3 were seeded into a six-well plate at 2 × 10^5^ cells per well. When reaching 80–90% confluence, ADSCs were replaced with the osteogenic (DMEM supplied with 10% FBS, 0.1 μM dexamethasone, 50 μM ascorbate-2-phosphate, and 10 mM β-glycerophosphate) and adipogenic (DMEM supplemented with 10% FBS, 0.5 mM isobutylmethylxanthine, 1 μM dexamethasone, 10 μM insulin, and 200 μM indomethacin) differentiation induction medium for induction culture. ADSCs replaced with the same amount of complete medium were set as a NC. Whether ADSCs underwent osteogenic differentiation or adipogenic differentiation was observed by Alizarin Red S staining or Oil Red O staining, respectively (19).

### Identification and Isolation of ADSC-Derived EVs

Upon reaching 80–90% confluence, ADSCs at passage 3 were rinsed with PBS and cultured for 48 h in serum-free endothelial cell growth medium (EGM)-2 MV (Lonza, Walkersville, MD, USA). The conditioned medium was collected and centrifuged to separate EVs, following the previously constructed protocols (20, 21). In brief, the conditioned culture medium was centrifuged at 300 g for 10 min, at 2,000 g for 10 min, and then at 10,000 g for 30 min to remove the remaining cells and cell fragments by using a 0.22-μm filter (Millipore, Billerica, MA, USA). After that, EVs (Optima L-90K ultracentrifuge; Beckman Coulter, Brea, CA, USA) were purified by 2-h ultracentrifugation at 100,000 g at 4°C. Finally, the EVs were separated by particle size fractionation and concentrated by centrifugation using a centrifuge tube (Millipore) with a molecular weight cut-off value of 100 kDa. The obtained EV particles were resuspended in PBS and stored at -80°C. Nanoparticle tracking analysis (NTA, Zeta View PMX 110, Particle Metrix, Meerbusch, Germany) and transmission electron microscope (TEM, JEOL microscope, JSM-7001TA, Tokyo, Japan) were implemented to analyze the diameter and ultrastructure of EVs. Western blot assay was performed to determine the surface protein markers CD9, CD63, and CD81 to identify EVs.

### Cell Source And Culture

Primary satellite cells (SCs) and SUI primary SCs were available from the Shanghai Cell Bank of the Chinese Academy of Sciences (Shanghai, China) and cultured in F12 medium supplemented with 10% FBS at 37°C with 5% CO_2_. Cells in the logarithmic phase were detached with 0.25% trypsin–0.2% EDTA solution and passaged in a ratio of 1:2. The SCs with good growth in the 4^th^ passage were used for subsequent experiments.

Primary fibroblasts and SUI primary fibroblasts were available from the Shanghai Cell Bank of the Chinese Academy of Sciences (Shanghai, China) and cultivated at 37°C in DMEM (Thermo Fisher Scientific, Waltham, Massachusetts, USA) containing 10% FBS (Thermo Fisher Scientific) until fibroblasts reached 80–90% confluence at passages 5 and 7.

### SC and Fibroblast Uptake of PKH26-Labeled ADSCs-EVs

The purified ADSCs-EVs were labeled for 5 min with 1 μM PKH26 (Sigma-Aldrich) at room temperature (22), rinsed with PBS, and centrifuged at 120,000g for 70 min to discard the unbound PKH26. Subsequently, the labeled pellet was resuspended in PBS/5% bovine serum albumin (BSA) and added to SC or fibroblast culture medium for 6 h. Twenty-four hours later, SCs or fibroblasts were rinsed with PBS and fixed with 4% paraformaldehyde. Afterwards, the cell nuclei were dyed with 4′,6-diamidino-2-phenylindole (Servicebio, Wuhan, China), and the SC cytoskeleton protein α-actin (Abcam, Cambridge, UK) and fibroblast cytoskeleton were stained with phalloidin (Sigma-Aldrich). Finally, a laser scanning confocal microscope (Zeiss, LSM710, Oberkochen, Germany) was utilized to observe the uptake of EVs by SCs or fibroblasts. In order to detect the transfer of miRNA from EVs to SCs or fibroblasts, SCs or fibroblasts stimulated by ADSCs-EVs for 6 h were collected, and the expression of miRNA was detected and analyzed by reverse transcription quantitative polymerase chain reaction (RT-qPCR).

### Grouping and Treatment of SCs or Fibroblasts

SCs or fibroblasts (2 × 10^5^) were seeded in six-well plates. After culturing for 24 h, cells were replaced with DMEM/F12 containing 10% EV-free FBS. Subsequently, SUI primary SCs or fibroblasts were divided into two groups, namely, SUI + GW4869 group (ADSC conditioned medium after GW4869 treatment; GW4869 (Sigma-Aldrich, St. Louis, MO) was used as an inhibitor of ADSC-EV secretion) (23, 24) and SUI + ADSCs-EVs group (ADSC conditioned medium with 30 μg ADSCs-EVs dissolved in 100 μl PBS). With 48-h treatment, cells were harvested for subsequent experiments. miR-93 mimics and miR-93 inhibitor plasmids (100 pmol, Genepharma Co., Ltd., Shanghai, China) were transfected into ADSCs with Lipofectamine 2000 (Thermo Fisher Scientific), incubated at 37°C for 4 h, and continued to culture in complete medium for 48 h. After the transfection efficiency was measured by RT-qPCR, EVs were isolated and acted on ADSCs (AD-EVs-NC and AD-EVs-inhibitor). Follow-up experiments were performed after 48 h. pcDNA-F3 or the corresponding NC plasmids (Genepharma) were transfected into SCs and fibroblasts by Lipofectamine 2000 and then treated for 48 h with 30 μg ADSCs-EVs or GW4869. After that, SCs of the above groups were harvested to analyze the expression of Pax7 and MyoD by immunofluorescence assay and Western blot assay; the fibroblasts were harvested to detect the expression of Elastin, Collagen I, and Collagen III by RT-qPCR and Western blot assay.

### Immunofluorescence Staining

The cells were rinsed three times with preheated PBS (10 min each time), fixed at room temperature with 4% formaldehyde for 30 min, permeabilized for 5 min with 0.2% Triton X-100, and blocked at room temperature for 30 min with 5% goat serum. Next, the cells were probed with primary antibody against Pax7 (ab187339, 1:100) (Abcam) or Myosin D (1: 50) (Cell Signaling Technology, Danvers, MA, USA) in a wet box at 4°C overnight. On the second day, the cells were re-probed with Alexa Fluor® 488- or Alexa Fluor® 594-labeled secondary antibody goat anti-rabbit IgG (ab150077 or ab150077, 1:200) (Abcam) in the darkroom. Lastly, cells were counterstained for 1 min with 1 μg/ml hoechst and then captured with a fluorescence microscope (DMI 6000, Leica Micro systems, Buffalo Grove, IL, USA).

### RT-qPCR

Total RNA was isolated from cells with TRIzol reagent (Invitrogen; Thermo Fisher Scientific), as per the manufacturer’s guidelines. SeraMir Exosome RNA Purification Kit (System Biosciences, Mountain View, USA) was used to isolate EV miRNA. For miRNA formation, 25 ng RNA of each sample was reversely transcribed using a universal cDNA synthesis kit (Exiqon, Woburn, MA), and then RT-qPCR was performed. The RT-qPCR reaction was performed with FastStart Universal SYBR Green Master Mix (Roche, Indianapolis, USA). The quantification analysis was calculated *via* the 2^-ΔΔCt^ method, where glyceraldehyde phosphate dehydrogenase and U6 were used for standardization, respectively. The primer sequences for genes are listed in **Table 1**.

**Table 1:** The primer sequence for genes.

Gene	Sequence
miR-93	Forward: 5’−AGTCTCTGGGCTGACTACATCACAG−3’
	Reverse: 5’−CTACTCACAAAACAGGAGTGGAATC−3’
F3	Forward: 5’-TTTCAGTGTTCAAGCAGTGATTCC-3′
	Reverse: 5’-CTACCGGGCTGTCTGTACTCATC-3′
Elastin	Forward: 5’-AGCTCCCTTGTTCTTGTGGA-3′
	Reverse: 5’-GGTGTGCCTAGCCAGACAGT-3′
Collagen 1	Forward: 5’-GAGGGCCAA GACGAAGACATC-3′
	Reverse: 5’-CAGATCACGTCATCGCACAAC-3′
Collagen III	Forward: 5’-AGCTGGACCAAAAGGTGATG-3′
	Reverse: 5’-GACCTCGTGCTCCAGTTAGC-3′
U6	Forward: 5’-GACGCACCAGAGCGAAAGC-3′
	Reverse: 5’-CCTCCGACTTTCGTTCTTGATT-3′
GAPDH	Forward: 5’−TCACCACCATGGAGAAGGC−3’
	Reverse: 5’−GCTAAGCAGTTGGTGGTGCA−3’

miR, microRNA; F3, coagulation factor III; GAPDH, glyceraldehyde phosphate dehydrogenase.

### Western Blot Assay

The cells were lysed with radioimmunoprecipitation assay buffer (Beyotime, Shanghai, China) containing 10 mM protease inhibitor (PMSF; Beyotime). Next, the protein lysate was loaded on 10% sodium dodecyl sulfate polyacrylamide gel electrophoresis, transferred onto a polyvinylidene fluoride membrane (Millipore), and then blocked for 1 h with 5% evaporated milk at room temperature. After that, the membrane was probed overnight at 4°C with primary antibody against F3 (ab104513, 1:1000), paired box gene 7 (Pax7) (ab199010, 1:1000), myosin D (MyoD) (ab203383, 1:1000), Elastin (ab217356, 1:500), Collagen I (ab34710, 1:2000), Collagen III (ab7778, 1:5000), β-Tubulin (ab6046, 1:2000), CD9 (ab92726, 1:2000), CD63 (ab134045, 1:2000), and CD81 (ab109201, 1:2000). Next, the membrane was re-probed with a horseradish peroxidase-conjugated antibody (1:4000, Servicebio) for 1 h at room temperature and captured by an ECL chemiluminescence detection kit (Servicebio) with the application for a BioSpectrum 600 Imaging System (UVP, California, USA). β-Tubulin antibody was utilized as a control of protein loading. Densitometry was carried out by ImageJ software (National Institutes of Health, Bethesda, MD, USA). An equal area was chosen inside each band, followed by the calculation of the mean of gray levels (in a 0–256 scale). Data were normalized to the values of background and of control β-Tubulin band (2, 25). The above primary antibodies were purchased from Abcam.

### Dual-Luciferase Reporter Gene Assay

Based upon the bioinformatics prediction, the sequences of F3-WT and F3-MUT containing the binding site of miR-93 were synthesized by Qijing Biological Technology Co., Ltd. (Hubei, Wuhan, China). F3-WT or F3-MUT was inserted into a pmirGLO luciferase vector (Promega, Madison, USA). The primary SCs or fibroblasts (1 × 10^5^) were seeded in a 12-well plate for 24 h, and the aforementioned luciferase reporter vectors with miR-93 mimic or its NC mimic were con-transfected into primary SCs or fibroblasts using Lipofectamine 3000 reagent (Invitrogen). Forty-eight hours post transfection, the luciferase activities were determined by a Dual-luciferase Reporter System (Promega). The experiment was repeated three times.

### Statistical Analysis

All *in vitro* experiments were conducted at least three times independently. SPSS 21.0 (IBM Corp., NY., USA) and GraphPad Prime 8.0 (GraphPad, USA) were utilized for the data analysis. The measurement data were presented as mean ± standard deviation. The differences between two groups were analyzed by Student’s t−test. The differences among multiple groups were analyzed using one-way analysis of variance (ANOVA). Tukey’s test was implemented for *post-hoc* multiple comparisons. *P* value less than 0.05 indicated a statistically significant difference.

## Results

### EVs Are Successfully Isolated From ADSCs

**Figure 1:**
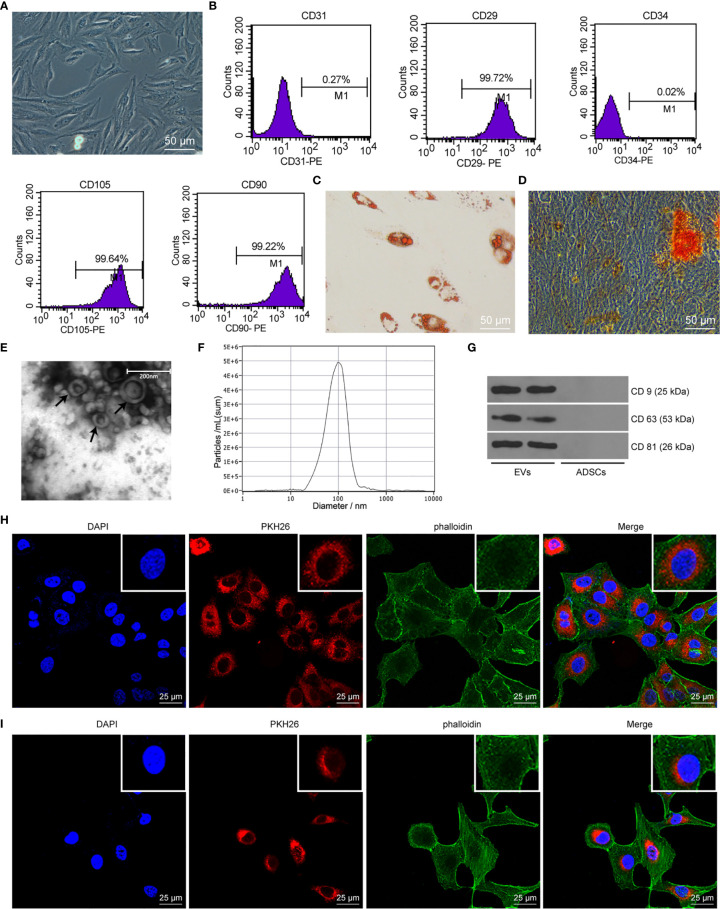
ADSC identification and characteristics of EVs. **(A)** Cell morphology of ADSCs at passage 3 was observed with an inverted microscope (× 200). **(B)** ADSC surface markers (CD31, CD34, CD29, CD90, and CD105) were analyzed by flow cytometry. **(C)** Oil red O staining of ADSCs cultured in adipogenic differentiation medium for 14 days (× 200). **(D)** Alizarin Red S staining of ADSCs cultured in osteogenic differentiation medium for 21 days (× 200). **(E)** Morphology of EVs (black arrow) observed by transmission electron microscopy (scale bar, 200 nm). **(F)** The particle distribution of ADSCs-EVs was measured with NTA (mean 106 ± 4 nm). **(G)** Western blot assay detection of CD9, CD63, and CD81 expression in ADSCs-EVs. **(H)** Laser scanning confocal images showed that the fibroblasts internalized PKH26-labeled EVs 24 h later (× 400), the nucleus was blue, the PKH26-EVs was red, and the cytoskeleton phalloidin was green. **(I)** Laser scanning confocal images showed that the SCs internalized PKH26-labeled EVs 24 h later (× 400), the nucleus was blue, the PKH26-EVs was red, and the cytoskeleton α-actin was green.

As previously reported, ADSCs-EVs protect SUI patients by increasing collagen synthesis in vaginal fibroblasts and reducing collagen degradation (26). However, the underlying mechanism remains unknown. In this study, we isolated ADSCs from rat adipose tissues, and ADSCs showed a typical cobblestone-like morphology (**Figure 1A**). As shown by flow cytometry, expression of ADSC markers CD29, CD90, and CD105 was positive, while the expression of vascular endothelial cell markers CD31 and CD34 was negative (**Figure 1B**). The results of Alizarin Red S staining or Oil Red O staining confirmed that ADSCs could differentiate into adipocytes and osteoblast types (**Figures 1C, D**). The above results show that the isolated ADSCs have the characteristics of MSCs. The EVs purified from the culture supernatant of ADSCs were characterized by a TEM and NTA. The results indicated that ADSCs-EVs were cup-shaped or round (**Figure 1E**), with an average vesicle diameter of (106 ± 4) nm (**Figure 1F**). Western blot assay suggested that the proteins CD63, CD81, and CD9 in ADSCs-EVs were positively expressed (**Figure 1G**). These data indicate that we successfully separated ADSCs-EVs. Laser scanning confocal images showed that the red fluorescent signal was basically detected in fibroblasts and SCs incubated with PKH26-labeled ADSCs-EVs for 24 h (**Figures 1H, I**), proving that EVs can be internalized by SCs and fibroblasts.

### ADSCs-EVs Regulate the Extracellular Matrix (ECM) Remodeling of SUI Primary Fibroblasts

**Figure 2:**
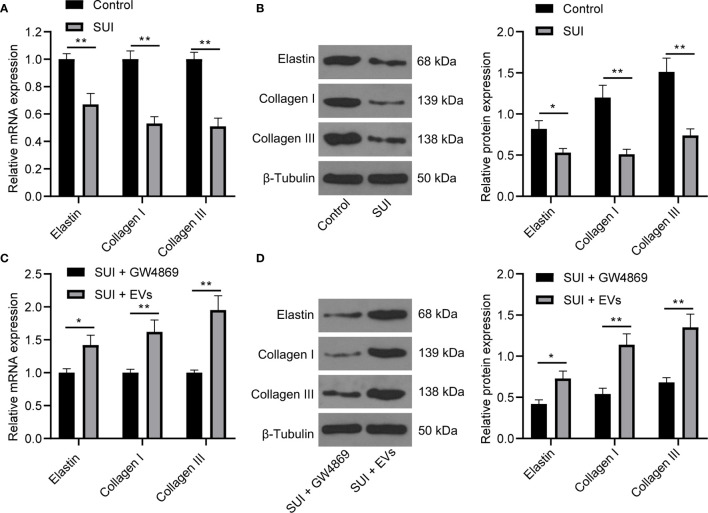
Effects of ADSCs-EVs on ECM remodeling of SUI primary fibroblasts. **(A, B)**. Expression levels of Elastin, Collagen I, and Collagen III in SUI primary fibroblasts determined by RT-qPCR and Western blot assay. **(C, D)**. Expression levels of Elastin, Collagen I, and Collagen III in SUI primary fibroblasts with ADSCs-EVs treatment determined by RT-qPCR and Western blot assay. GAPDH was used as the mRNA reference gene in the RT-qPCR data, and 2^−ΔΔCt^ was used for quantitative analysis; the Western blot assay data was normalized to the background value and the control β-Tubulin band value. N = 3. The differences between two groups were analyzed by Student’s t−test **(A–D)**. *P < 0.05; **P < 0.01.

There are articles suggesting that changes in the composition of the ECM are one of the main causes of SUI. This change is mainly manifested in the decrease of elastin fibers and collagen fibers, and the disorder of elastin fibers (22, 27). We first found by RT-qPCR and Western blot assay that the expression levels of Elastin, Collagen I, and Collagen III in SUI primary fibroblasts were lower than those of primary fibroblasts (**Figures 2A, B**, all *P* < 0.05). For figuring out the therapeutic effect of ADSCs-EVs *in vitro*, we used the extracted EVs for direct research after referring to the latest exosome research guide “MISEV2018”, that is, SUI primary fibroblasts were treated with ADSCs-EVs for 48 h, with GW4869 treatment as a control. We found in RT-qPCR and Western blot assay that expression levels of Elastin, Collagen I, and Collagen III were increased after treatment with ADSCs-EVs (**Figures 2C, D**, all *P* < 0.01). It shows that ADSCs-EVs can regulate the ECM remodeling of SUI primary fibroblasts.

### ADSCs-EVs Promote the Activation of SUI Primary SCs

**Figure 3:**
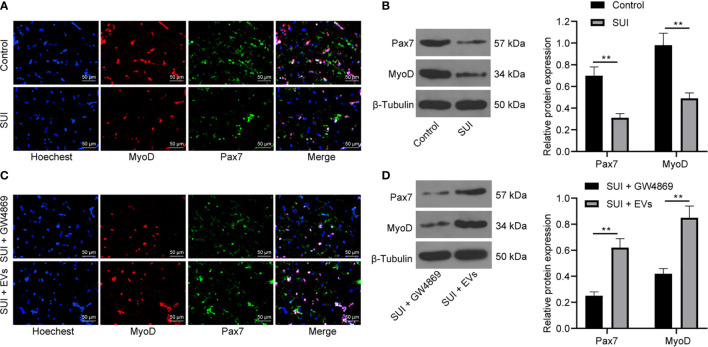
Effects of ADSCs-EVs on activation of SUI primary SCs. **(A, B)** Immunofluorescence assay **(A)** and Western blot assay **(B)** were used to detect the influence of Pax7 and MyoD protein expression in SUI primary SCs. **(C, D)** Immunofluorescence assay **(C)** and Western blot assay **(D)** were used to detect the effects of ADSCs-EVs on Pax7 and MyoD protein expression in SCs. The Western blot assay data was normalized to the background value and the control β-Tubulin band value. N = 3. The differences between two groups were analyzed by Student’s t−test **(B, D)**. **P < 0.01.

SC activation is related to the recovery of muscle tissue damage. Pax7 is a specific marker of adult SCs. The activation of MyoD helps expand resting myogenic SCs (28, 29). We first confirmed by immunofluorescence assay and Western blot assay that the expression of Pax7 and MyoD in SUI primary SCs was decreased versus primary SCs (**Figures 3A, B**, both *P* < 0.01). For figuring out the therapeutic effect of ADSCs-EVs *in vitro*, SUI primary SCs were treated with ADSCs-EVs for 48 h, with GW4869 treatment as a control. We found in immunofluorescence assay and Western blot assay that expression of Pax7 and MyoD was increased after treatment with ADSCs-EVs (**Figures 3C, D**, both *P* < 0.01). It shows that ADSCs-EVs can promote the activation of SUI primary SCs.

### ADSCs-EVs Secrete miR-93 to Regulate the ECM Remodeling of Fibroblasts and Promote the Activation of SCs

**Figure 4:**
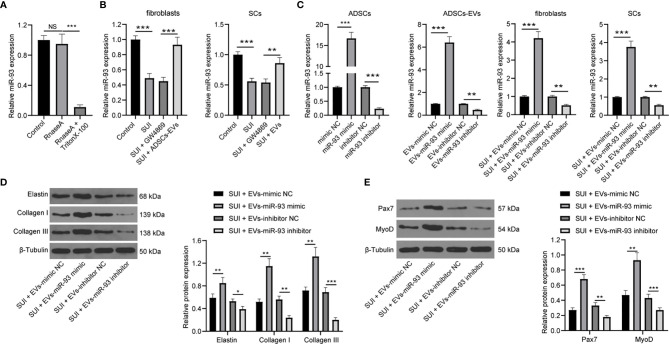
ADSCs-EVs regulate fibroblasts ECM remodeling and SC activation through miR-93. **(A)** RT-qPCR for detecting miR-93 expression in culture medium after the addition of RNase A alone or the combination of RNase A and Triton X-100 to the culture medium of ADSCs. **(B)** RT-qPCR detection of miR-93 expression in fibroblasts and SCs. **(C)** RT-qPCR for detecting miR-93 expression after miR-93 mimic or inhibitor intervention (ADSCs were transfected with miR-93 mimic or inhibitor, and then the isolated EVs were utilized to act on SUI fibroblasts or SCs). **(D)** Western blot assay for determining the influences of Elastin, Collagen I, and Collagen III protein expression in SUI primary fibroblasts in each group. **(E)** Western blot assay for determining the influences of Pax7 and MyoD protein expression in SUI primary SCs in each group. U6 was used as the miR-93 reference gene in the RT-qPCR data, and 2^−ΔΔCt^ was used for quantitative analysis; the Western blot assay data was normalized to the background value and the control β-Tubulin band value. N = 3. The differences between two groups were analyzed by Student’s t−test **(B–E)** The differences among multiple groups were analyzed using one-way ANOVA and Tukey’s *post hoc* test **(A)** NS, not significant. *P < 0.05; **P < 0.01; ***P < 0.001.

Evidence has shown that ADSCs-EVs can carry miR-93 (12), and miR-93 can regulate ECM remodeling in SUI fibroblasts (13). Therefore, we speculated that miR-93 encapsulated in ADSCs-EVs might regulate ECM remodeling in SUI fibroblasts. First, we tested the expression of miR-93 in the culture medium of ADSCs. The addition of RNase A to the culture medium of ADSCs hardly changed the expression of miR-93, but it was significantly reduced upon treatment with RNase A + Triton X-100 (**Figure 4A**), which indicated that the extracellular mir-93 is mainly enveloped in the membrane rather than directly released. Next, RT-qPCR was implemented to detect miR-93 expression in fibroblasts and SCs, and it was found that miR-93 expression was reduced in SUI fibroblasts and SCs in contrast to that in primary fibroblasts and SCs, and miR-93 expression was elevated in ADSCs-EVs in comparison to ADSCs treated with GW4869 (all *P* < 0.001; **Figure 4B**). Combined with the results of **Figures 1H, I**, it shows that ADSCs-EVs can carry miR-93 into fibroblasts and SCs.

To further confirm the role of EVs-miR-93 in SUI, we transfected miR-93 mimic and miR-93 inhibitor into ADSCs, and verified the transfection of miR-93 mimic and inhibitor by RT-qPCR. Afterwards, ADSCs-EVs were extracted and miR-93 expression was detected. It was found that miR-93 mimic elevated miR-93 expression in the EVs, while miR-93 inhibitor reduced miR-93 expression in the EVs. Then, SUI fibroblasts and SCs were treated with ADSCs-EVs. It was suggested that SUI-EVs-mimic elevated miR-93 expression, while SUI-EVs-inhibitor reduced miR-93 expression in cells (**Figure 4C**). Based on the results of Western blot assay, we found that SUI-EVs-mimic contributed to increased expression levels of Elastin, Collagen I, and Collagen III in fibroblasts and increased expression levels of Pax7 and MyoD in SCs. Treatment of SUI-EVs-inhibitor exhibited an opposite trend (**Figures 4D, E**). The above results indicate that EVs secreted by ADSCs are delivered to fibroblasts through miR-93, thereby regulating the ECM remodeling in fibroblasts and promoting the activation of SCs.

### ADSCs-EVs Secrete miR-93 by Downregulating F3 Expression

**Figure 5:**
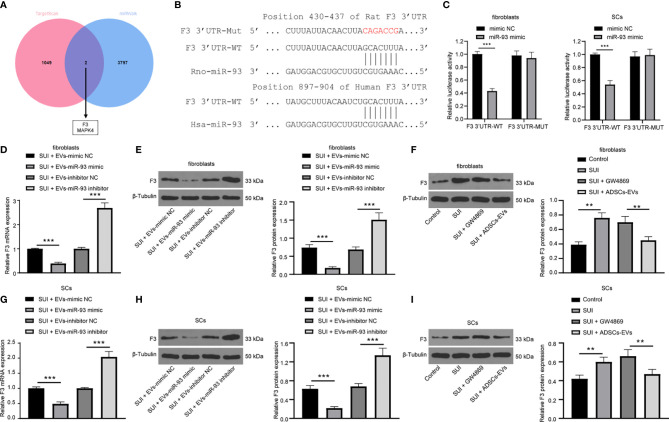
miR-93 target F3 in EVs. **(A)** TargetScan and miRWalk for predicting the Venn map of the downstream target genes of miR-93. **(B)** TargetScan for predicting the binding sites of miR-93 and F3 3’UTR. **(C)** Dual-luciferase reporter gene assay on the targeting relationship between miR-93 and F3 in primary fibroblasts and SCs. **(D, E)** RT-qPCR **(D)** and Western blot assay **(E)** were utilized to detect the mRNA and protein expression of F3 in ADSCs-EVs-treated SUI fibroblasts with the interference of miR-93 mimics or inhibitors. **(F)** Western blot assay was utilized to detect the protein expression of F3 in ADSCs-EVs-treated SUI fibroblasts. **(G, H)** RT-qPCR **(G)** and Western blot assay **(H)** were utilized to detect the mRNA and protein expression of F3 in ADSCs-EVs-treated SUI SCs with the interference of miR-93 mimics or inhibitors. **(I)** Western blot assay was utilized to detect the protein expression of F3 in ADSCs-EVs-treated SUI SCs. U6 and GAPDH were used as the miR-93 and F3 reference genes in the RT-qPCR data, and 2^−ΔΔCt^ was used for quantitative analysis; the Western blot assay data was normalized to the background value and the control β-Tubulin band value. N = 3. The differences between two groups were analyzed by Student’s t−test **(C, D, E, G, H)**. The differences among multiple groups were analyzed using one-way ANOVA and Tukey’s *post hoc* test **(F, I)**. **P < 0.01; ***P < 0.001.

With the aim to continue to reveal the downstream molecular mechanism of miR-93 involved in the treatment of SUI with ADSCs-EVs, the online databases TargetScan (http://www.targetscan.org/vert_71/) and miRWalk (http://mirwalk.umm.uni-heidelberg.de/) were searched to predict the downstream target genes of miR-93, and 1,051 and 3,799 downstream genes were obtained, respectively. The Venn diagram was plotted with an online website (http://jvenn.toulouse.inra.fr/app/example.html), and from the intersection, we found two common downstream genes (F3 and MAPK4) (**Figure 5A**). Besides, an article has indicated that miR-93 directly targets F3 to participate in the pathogenesis of leiomyomas (16). Predictions from TargetScan found that there were binding sites in the 3’UTR of miR-93 and F3 in both humans and rats (**Figure 5B**). Dual-luciferase reporter gene assay indicated that the miR-93 mimics + F3 3’UTR-WT group had a significant decrease in luciferase activity versus the mimic-NC+ F3 3’UTR-WT group (*P* < 0.001). There was no change in luciferase activity between the two mutant groups (*P* > 0.05; **Figure 5C**). In addition, after miR-93 mimics or inhibitors intervened with ADSCs-EVs-treated SUI fibroblasts, RT-qPCR and Western blot assay were implemented to detect the F3 expression. miR-93 mimics downregulated F3 expression, while miR-93 inhibitors upregulated F3 expression in SUI fibroblasts (**Figures 5D, E**). Meanwhile, to further confirm that miR-93 secreted by ADSCs-EVs could inhibit F3 expression in fibroblasts, we treated SUI primary fibroblasts with ADSCs-EVs or GW4869. Western blot assay was utilized to detect F3 protein expression. We found that F3 protein expression in SUI fibroblasts was upregulated; P3 protein expression was reduced in ADSCs-EVs-treated SUI fibroblasts (**Figure 5F**). This regulatory role also existed in SCs (**Figures 5G–I**). The above results indicate that miR-93 carried by ADSCs-EVs can inhibit F3 expression in fibroblasts and SCs.

### Overexpression of F3 Reverses the Effects of ADSCs-EVs on ECM Remodeling of SUI Primary Fibroblasts and SC Activation

**Figure 6:**
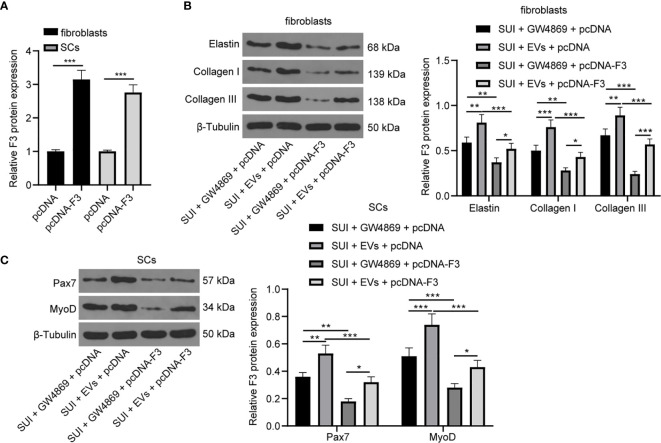
ADSCs-EVs reverse the effect of F3 on SUI fibroblasts and SCs. **(A)** RT-qPCR detection of the transfection efficiency of F3. **(B)** Western blot assay was utilized to detect the expression levels of Elastin, Collagen I, and Collagen III in SUI fibroblasts in each treatment group. **(C)** Western blot assay was utilized to detect the expression levels of Pax7 and MyoD in SUI SCs in each treatment group. GAPDH was used as the mRNA reference gene in the RT-qPCR data, and 2^−ΔΔCt^ was used for quantitative analysis; the Western blot assay data was normalized to the background value and the control β-Tubulin band value. N = 3. The differences between two groups were analyzed by Student’s t−test **(A)**. The differences among multiple groups were analyzed using one-way ANOVA and Tukey’s *post hoc* test **(B, C)**. *P < 0.05; **P < 0.01; ***P < 0.001.

We have previously confirmed that ADSCs-EVs could carry miR-93 into fibroblasts and SCs to regulate their functions, and miR-93 secreted by ADSCs-EVs could inhibit the expression of F3 in fibroblasts. Finally, in order to explore whether overexpression of F3 could reverse the effects of ADSCs-EVs on the ECM remodeling of SUI primary fibroblasts and the activation of SCs, we transfected pcDNA-F3 or pcDNA into fibroblasts and SCs, respectively, through Lipofectamine 2000, and then treated with 30 μg ADSCs-EVs or GW4869 for 48 h. The RT-qPCR detection of transfection efficiency found that F3 expression was significantly increased in fibroblasts or SCs treated with pcDNA-F3 (**Figure 6A**). According to the results of Western blot assay, we found that expression levels of Elastin, Collagen I, and Collagen III in SUI fibroblasts were elevated after ADSCs-EVs treatment, and the expression levels of which were reduced upon treatment of ADSCs-EVs and F3 overexpression (**Figure 6B**). Similarly, expression levels of Pax7 and MyoD in SUI SCs were elevated after ADSCs-EVs treatment, and the expression levels of which were reduced upon treatment of ADSCs-EVs and F3 overexpression (**Figure 6C**). This shows that overexpression of F3 can reverse the effects of EVs-treated ADSCs on the ECM remodeling of fibroblasts and the activation of SCs.

## Discussion

Recently, cell-based therapy for SUI has been implemented both experimentally and clinically (30, 31). Among which, ADSCs show several merits for clinical application, such as less invasive harvesting procedure, ease of access to enough adipose tissue, and the ability for myogenic differentiation (32, 33). ADSCs have been implied to exert function in a paracrine manner *via* promoting angiogenesis and stimulating surrounding cells *in vivo* trials (34). In this study, ADSCs-EVs were co-cultured with SUI primary SCs and fibroblasts, and the *in vitro* therapeutic effects of ADSCs-EVs were observed. We further explored whether miR-93 released by ADSCs-EVs could play a therapeutic role in SUI rats by inhibiting F3 expression from the aspects of regulating SC activation and fibroblast ECM remodeling.

The change in ECM composition in the urethral sphincter is a significant pathogenesis of SUI, and this change is presented with reduced elastin fibers and collagen fibers, and disordered alignment of elastin fibers (27, 35). Several articles have elucidated that stem cells are able to motivate the ECM remodeling to promote the urethral function recovery in SUI (36, 37). In our present study, ADSCs-EVs were found to regulate ECM remodeling in SUI primary fibroblasts, reflecting by enhanced expression of Elastin, Collagen I, and Collagen III in SUI primary fibroblasts. Similarly, Zhang et al. have found that BMSCs-EVs enhance the synthesis of ECM proteins (Elastin, Collagen I, and Collagen III) and restore the thickness and length of elastic and collagen fibers (22). In accordance with our findings, ADSCs-conditioned medium has been reported to modulate collagen metabolism in the SUI fibroblasts through enhancing collagen synthesis and reducing collagenolysis (26) and ADSCs-EVs could be internalized by fibroblasts, further promoting the collagen synthesis (38). All these evidences reveal that ADSCs-EVs regulates ECM remodeling of SUI fibroblasts.

The processes of activation of SCs are related to recovery of muscle tissue injuries, indicating their suitable application for SUI treatment (28). Upon SC activation, the expression of MyoD is elevated, while Pax7 is decreased (39, 40). Pax7 is a marker specific for SC adult muscle (41), and MyoD is important for the SC differentiation and MyoD activation helps to amplify the quiescent myogenic SCs (42). A prior study has demonstrated that elevated Pax7 in adult primary myoblasts suppresses MyoD expression, thereby suppressing myogenesis (43), implying that upregulated MyoD and downregulated Pax7 are indicative of activated SCs. In this current study, we confirmed that the expression of Pax7 and MyoD in SUI primary SCs was decreased versus primary SCs, and ADSCs-EVs can promote the activation of SUI primary SCs. Similarly, urine-derived stem cell-derived exosomes could promote the SC activation, thereby enhancing the recovery of the injured pubococcygeus muscle and alleviating the symptoms of SUI (28). Nevertheless, the function of ADSCs-EVs in SC activation in SUI needs further verification.

Due to the lack of effective delivery methods, the progress of miRNA treatment is hindered. To address this problem, EVs have been used to deliver exogenous miRNA and mRNA to target cells (19). Evidence has shown that ADSCs-EVs can carry miR-93 (12), and miR-93 can regulate ECM remodeling in SUI fibroblasts (13). Therefore, we speculated that miR-93 encapsulated in ADSCs-EVs might regulate ECM remodeling in SUI fibroblasts and SC activation. It has been reported that miR-93-5p acts as a non-invasive biomarker in detecting cisplatin-stimulated proximal tubular injury (44). Also, miR-93-3p has been considered as a diagnostic biomarker of acute kidney injury (AKI) in patients (45). As for the functions of miR-93 encapsulated in ADSCs-EVs, a study has suggested that miR-93-5p-enhanced ADSC-EVs are capable of preventing cardiac injury *via* hindering autophagy and the inflammatory response (12). Another article has indicated that ADSC-EVs carrying miR-93-5p could serve as a novel therapeutic target for the treatment of sepsis-induced AKI (12). With the aim to continue to reveal the downstream molecular mechanism of miR-93 involved in the treatment of SUI with ADSCs-EVs, the online databases predicted the binding site between miR-93 and F3. Besides, an article has indicated that miR-93 directly targets F3 to participate in the pathogenesis of leiomyomas (16). Thus, we found that miR-93 carried by ADSCs-EVs could inhibit F3 expression in fibroblasts and SCs. In line with our findings, Yang et al. have found that upregulated miR-93 elevates expression of collagen through targeting calpain-2, which clarifies the role of miR-93 and its target gene, and offers a theoretical basis SUI treatment (13). However, the targeting relation between miR-93 and F3 in SUI should be confirmed in future research.

**Figure 7:**
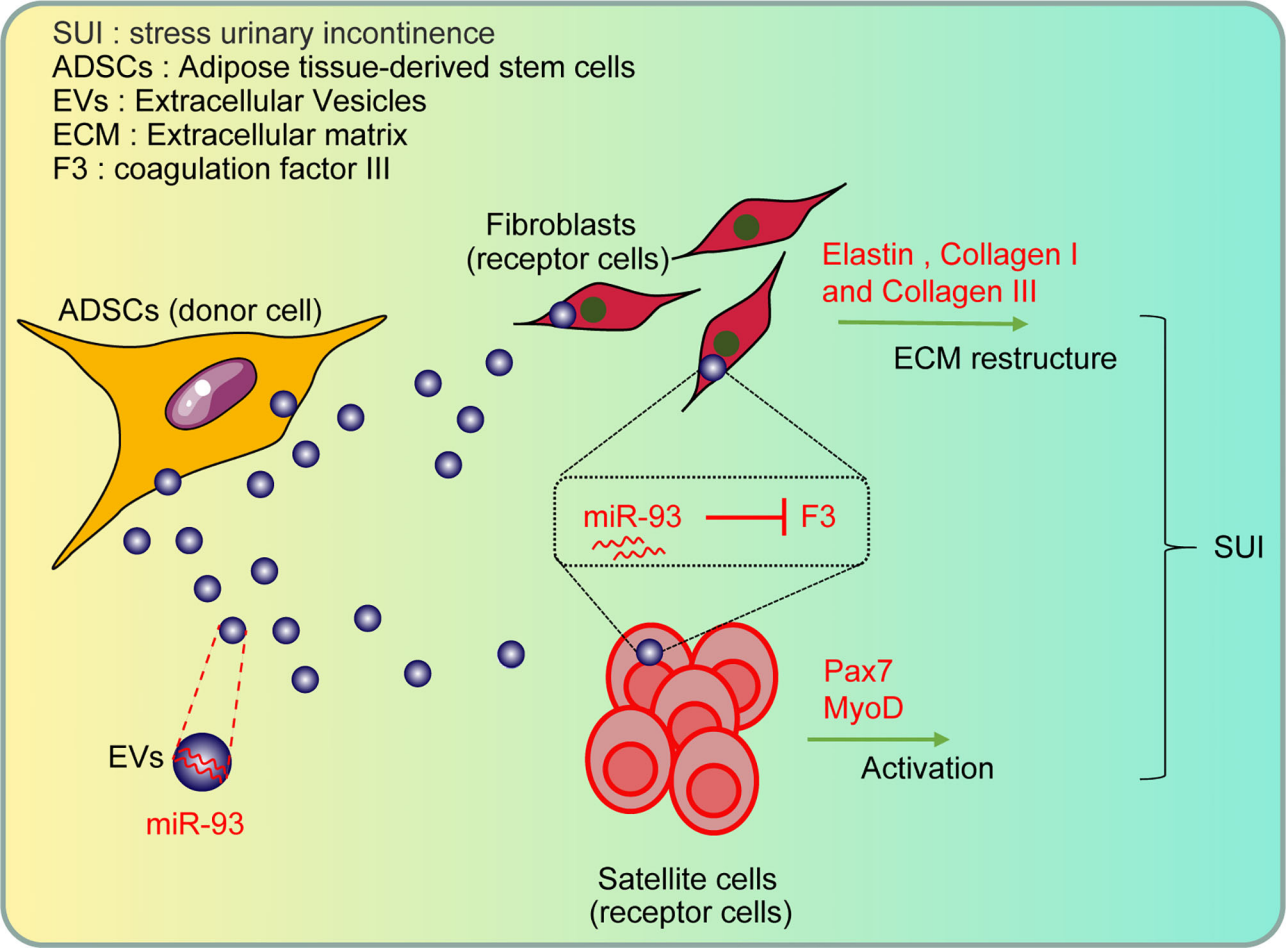
The role and mechanism of miR-93 delivered by ADSCs-EVs in the treatment of SUI *in vitro* through regulating the target gene F3: the ADSCs-EVs can be endocytosed by fibroblasts and SCs, and then the carried miR-93 can enter the recipient cells to upregulate the expression of miR-93 in cells, inhibit the expression of F3 (target gene of miR-93), and then regulate the ECM remodeling of fibroblasts and the activation of SCs, playing a role in the treatment of SUI *in vitro*.

Collectively, our study provides evidence that miR-93 delivered by ADSCs-EVs could regulate ECM remodeling of SUI fibroblasts and promote activation of SUI SCs through targeting F3 (**Figure 7**). Therefore, this study provides a basis for SUI treatment *in vivo* with miR-93 from ADSCs-EVs. However, further investigations are still required to figure out the specific mechanism of ADSCs-EVs by which the miR-93/F3 axis is involved in SUI. In addition, whether other types of MSC-derived EVs have a role in SUI will be further discussed in our future research. According to the previous research methods, the target cells were mainly co-cultured with ADSCs and then co-cultured with GW4869 to confirm the role of ADSCs. In this study, we used the extracted EVs for direct research after referring to the latest exosomes research guide “MISEV2018”. We used the ADSC-conditioned medium treated with ADSC-EVs and the ADSC-conditioned medium treated with GW4869 as a control to directly act on target cells to explore the effects of ADSC-EVs. The two methods may be different, and we will change some other methods in the future to verify the role and mechanism of miR-93 delivered by ADSCs-EVs and its target gene F3 in the *in vitro* therapy of SUI, such as co-culturing the target cells with ADSCs, followed by the co-cultivation with GW4869 to confirm the role of ADSCs.

## Data Availability Statement

The original contributions presented in the study are included in the article/supplementary material. Further inquiries can be directed to the corresponding author.

## Ethics Statement

The animal study was reviewed and approved by Zhengzhou Central Hospital Affiliated to Zhengzhou University.

## Author Contributions

LW contributed to the study concepts, study design, and definition of intellectual content. LW, YW, YX, and JPM contributed to the literature research. LW and YW contributed to the manuscript preparation and HL contributed to the manuscript editing and review. HZ, JD, YH, YY, and JRM contributed to the experimental studies and data acquisition. LW, HL, YW, YX, and JPM contributed to the data analysis and statistical analysis. All authors contributed to the article and approved the submitted version.

## Conflict of Interest

The authors declare that the research was conducted in the absence of any commercial or financial relationships that could be construed as a potential conflict of interest.

## Publisher’s Note

All claims expressed in this article are solely those of the authors and do not necessarily represent those of their affiliated organizations, or those of the publisher, the editors and the reviewers. Any product that may be evaluated in this article, or claim that may be made by its manufacturer, is not guaranteed or endorsed by the publisher.
